# Microwave-Assisted Synthesis of Highly-Crumpled, Few-Layered Graphene and Nitrogen-Doped Graphene for Use as High-Performance Electrodes in Capacitive Deionization

**DOI:** 10.1038/srep17503

**Published:** 2015-12-08

**Authors:** Ahmad Amiri, Goodarz Ahmadi, Mehdi Shanbedi, Maryam Savari, S. N. Kazi, B. T. Chew

**Affiliations:** 1 Department of Mechanical Engineering, University of Malaya, Kuala Lumpur, Malaysia; 2 Department of Mechanical and Aeronautical Engineering, Clarkson University, Potsdam, NY 13699, USA; 3 Department of Chemical Engineering, Faculty of Engineering, Ferdowsi University of Mashhad, Mashhad, Iran; 4 Faculty of Computer Science and Information Technology, University of Malaya, Kuala Lumpur, Malaysia

## Abstract

Capacitive deionization (CDI) is a promising procedure for removing various charged ionic species from brackish water. The performance of graphene-based material in capacitive deionization is lower than the expectation of the industry, so highly-crumpled, few-layered graphene (HCG) and highly-crumpled nitrogen-doped graphene (HCNDG) with high surface area have been introduced as promising candidates for CDI electrodes. Thus, HCG and HCNDG were prepared by exfoliation of graphite in the presence of liquid-phase, microwave-assisted methods. An industrially-scalable, cost-effective, and simple approach was employed to synthesize HCG and HCNDG, resulting in few-layered graphene and nitrogen-doped graphene with large specific surface area. Then, HCG and HCNDG were utilized for manufacturing a new class of carbon nanostructure-based electrodes for use in large-scale CDI equipment. The electrosorption results indicated that both the HCG and HCNDG have fairly large specific surface areas, indicating their huge potential for capacitive deionization applications.

Desalination has been considered as one of the promising methods for supplying fresh water from seawater and brackish water by absorbing minerals. Although about 71% of the Earth’s surface is covered by water, only 3% is fresh water, and salt-water oceans and seas contain about 97% of the world’s water. To date, desalination has not played a key role for supplying potable water, and it just accounts for a very small fraction^1^,^2^. It is obvious that the desalination of seawater can be considered as a promising approach to solve the need for fresh water^3^. Numerous traditional approaches, such as reverse osmosis (RO), electrodialysis, multiple-effect distillation, and multi-stage flash and vapor-compression distillation have been used to supply fresh water. Multi-stage flash and multiple-effect distillation as the thermal approaches are energy-intensive and rather expensive^1^,^4^. RO, however, is an energy-efficient approach for desalination, which recently was used in a commercial plant and achieved a record of 1.8 kWh/m^3^^2^. RO also is one of the largest volume applications for membrane technology, and most new installations for seawater desalination will use (or at least consider) RO technology.

However, despite improvements in different approaches and the easy accessibility of seawater, most of the desalination methods have not achieved a great deal of success in terms of water treatment, implying the need for more effective and economical methods.

Numerous innovative approaches have been applied to decrease the cost of seawater desalination^5^. One new promising method is electrosorption with porous electrodes that was introduced as a suitable route for eliminating different ions from aqueous media as well as suspensions^6^. Interestingly, electrosorption can operate at low direct voltages and powers, which distinguishes this method from other suggested methods. Also, electrosorption generates no secondary pollution, which is common in other conventional techniques such as thermal approaches, RO, and distillation columns^7^.

The recently-improved electrosorption can remove charged ionic species from seawater as a cost-effective and energy-efficient method^8^,^9^,^10^. The fundamental theory of electrosorption comprises making the ions move toward oppositely polarized electrodes via applying an electric field^7^. By placing the electrodes with appropriate surface area and high electrical conductivity into the direct electrostatic field, they easily can generate an intense electrical double layer for absorbing charged ions from solutions 7,9,11. More beneficially, as the electric field is eliminated, the ions absorbed on the surface of electrodes are effortlessly emancipated^11^. Its characteristics of low energy consumption, being an environmentally-friendly process without harsh chemicals, easy regeneration of electrodes, and the cleaning process make it attractive for use in the field of water treatment. To enhance the electrosorptive procedure, highly-porous materials, such as carbon nanotubes, carbon fibers, carbon aerogels, and graphene, are highly-desired materials for use in electrosorptive electrodes or capacitors^11^,^12^. High conductivity, appropriate pore size distribution, and specific surface area are the essential factors for realization of high-performance water-treatment procedures with the use of carbon nanostructures^5^,^6^,^12^.

Recently, graphene, due to its many promising properties, has been introduced as a unique material and has received considerable attention in the scientific community^13^,^14^,^15^,^16^,^17^,^18^. In particular, large specific area, outstanding tensile modulus, and high electrical conductivity at room temperature make graphene an ideal material for electrosorption and for the fabrication of electrosorption electrodes^19^. The suitability of graphene for NaCl electrosorption from aqueous suspensions recently was suggested in several studies^9^,^10^,^11^,^20^,^21^,^22^,^23^,^24^. Also, the specific electrosorptive capacity of graphene is about 23.18 μmol/g, which is quite significant^11^. Developing an economical and industrially-scalable method for synthesizing graphene, however, is critical for its large-scale applications.

It is well known that electrosorption capacity improves as the surface area of carbon nanostructures increases. Actually, the large electrosorptive capacity of graphene definitely is highly dependent on the geometry, level of charge separation at the interface, and surface properties of the electrode materials^8^. Therefore, to manufacture high-performance electrodes for electrosorptive applications, materials with high surface areas, such as crumpling graphene, in particular few-layered graphene, can be more suitable. Crumpling the 2D graphene and nitrogen-doped graphene (NDG) with chemical treatment can be an approach to achieve materials with high surface area and pore structures. Thus, highly-crumpled, few-layered, nitrogen-doped graphene and pure graphene with large surface area seem to have these properties. Also, NDG is promising material due to the desirable pseudo-capacitance obtained from the quick Faradic redox reactions among the nitrogen groups and the ions in the electrolytes^20^,^25^. Furthermore, the presence of nitrogen in the graphene frameworks could increase the electrical conductivity^26^. Nitrogen doping can also increase the wettability of the interface between the electrolyte and electrodes^27^. Xue *et al.*^20^ showed the specific capacitance of NDG to be much higher than that of pristine graphite. Three reason for having higher specific capacitance, i.e., increased specific surface area^11^, increased pseudo-capacitive effect of electrodes by nitrogen doping and improving the wettability of the interface between the electrolyte and the electrode^27^, were introduced for NDG. Thus, the combination of highly-crumpled graphene and nitrogen doping can be effective novelties for increasing the specific capacitance of electrodes.

To this end, a promising and efficient procedure was developed to fabricate highly-crumpled, few-layered graphene (HCG) and nitrogen-doped graphene (HCNDG) nanosheets with large surface area. Also, the method of synthesizing HCG and HCNDG seems to be novel, simple, cost-effective, and industrially scalable. HCG and HCNDG, due to their unique specific surface areas, provide significant improvement in terms of electrosorptive performance.

## Material and Methods

### Synthesizing HCG and HCNDG

Figure S1 shows the schematic procedure of exfoliation of graphite flakes to HCG and HCNDG with cyanamide. The mechanism for the exfoliation of the graphite to HCG and HCNDG includes the generation of semi-stable diazonium ions, which then initiate a radical reaction with the flakes. Then, treated graphite with higher dispersibility in dimethylformamide (DMF) opens a new gateway for expanding graphite to graphene flakes by placing between layers. Pristine graphite and deionized water were poured into a vessel. The reaction vessel was sonicated after adding cyanamide and isoamyl nitrite, and the contents were poured into a Teflon vessel for performing the reaction under microwave irradiation. Then, the mixture was mixed vigorously, and, after cooling to room temperature, it was filtered and washed. Thus, a semi-stable diazonium ions were produced, resulting in a radical reaction with the graphite flakes. The high-dispersibility product, due to functional group, was sonicated for 1 hr in DMF. The resulting black ink-like dispersion was left to sit for 24 hr to separate the large, unstable graphite aggregates. The dispersed cyanamide-treated graphene was collected using low-speed centrifugation. The centrifuged supernatant was dried. The resulting HCG, comprised of graphene flakes, was stored for further treatment. Then, to prepare HCNDG, the resulting HCG materials were placed in a furnace under a nitrogen atmosphere and heated to 900 °C to change the functionality, including the amine group, into nitrogen-containing species, such as C_2_N_2_^+^, C_3_N_2_^+^, and C_3_N_3_^+^^28^,^29^. This process provides nitrogen sources for changing HCG into HCNDG.

### Preparation of HCG and HCNDG-Based Electrodes

**Figure 1:**
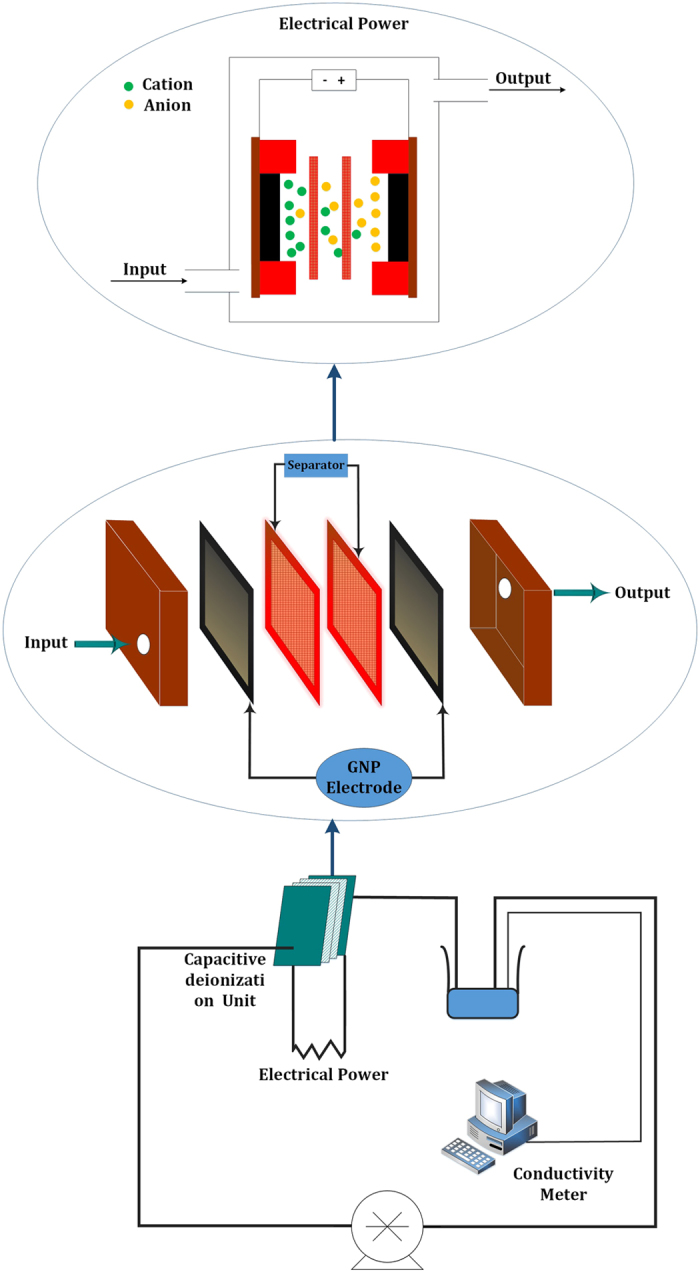
Schematic of (1) the electrosorptive unit and (2) the cell batch-mode experiment with HCG or HCNDG electrodes.

To manufacture the HCG and HCNDG-based electrodes, the prepared HCG or HCNDG powder and polytetrafluoroethylene (PTFE) as binder were used in the weight ratio of 92% and 8%, respectively^11^. To achieve good adhesion, the pristine mixture of HCG, HCNDG, and PTFE powders was ground for 3 hr. Ethanol (around10 mL) was added dropwise to the mixture to increase the moisture of the samples, and, then, the mixture was pressed on a graphite sheet. Each HCG-based and HCNDG-based electrode was 70 mm wide, 140 mm long, and 0.5 mm thick. The HCG-based and HCNDG-based electrodes were placed into a capacitive deionization instrument for further investigation, as shown schematically in Fig. 1.

### Batch Mode Electrosorptive Experiment

Batch adsorption experiments were performed in a recycling system to investigate the electrosorptive capacity of HCG-based and HCNDG-based electrodes. Figure 1 shows that the electrosorption unit cell consist of a power supply, a peristaltic pump, and a conductivity meter (DDS-307), which were used to prepare aforementioned recycling system. A peristaltic pump was utilized to feed the solution continuously into the cycle. The volumetric flow rate and temperature of the solution were selected based on previous studies^9^ for optimal performance, which were 25 ml/min and 20 °C, respectively. In detail, the CDI unit consists of two symmetrical rectangular pieces of silicon with a space defined by a gasket between them. The gasket size was 4 mm, and the distance between the electrodes was 3 mm. The silicon plates have fluidic inlet and outlet ports with the same diameter of 4 mm. Also, a direct voltage of 1.0 to 2.0 V was used, and the solution’s temperature was kept at 25 °C. In each experiment, the conductivity’s variation in the NaCl solution was monitored continuously and measured at the outlet using an ion conductivity meter. The resulting solution had an initial conductivity of about 51 μS/cm. An ion conductivity meter was used to measure the change in the concentration of the NaCl solutions at the outlet of the unit cell. To obtain the concentration of NaCl in different solutions during the experiments, a relationship between conductivity and concentration was introduced at the above-mentioned conditions.

Figure S2 illustrates the correlation that results from the relationship of the solution’s conductivity (μs/cm) and its concentration (mg/L). The linear equation obtained from the experimental results is shown by Eq. 1:


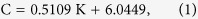


where C and K represent the solution’s concentration(mg/L) and its conductivity (μS/cm), respectively. Here, the regression coefficient (R^2^) of Eq. (1) is 0.99. The correlation of Li *et al.*^9^ is reproduced in Figure S2 for comparison. This figure shows that the present data and the correlation given by Eq. (12) are in good agreement with the correlation suggested in^9^.

Also, the electrosorptive capacity (amount of NaCl adsorbed per unit mass of the HCG-based and HCNDG-based electrodes) can be calculated using^9^:


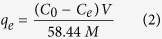


where q_e_ is the electrosorptive capacity (μmol/g), C_0_ and C_e_ are the initial and final concentration of NaCl (mg/L), respectively, V is the volume of the solution in the cycle (50 mL), and M is the mass of the HCG-based or HCNDG-based electrodes. Characterization instruments and methods are described in detail in the Supplementary Information.

## Result and Discussion

First, highly-crumpled graphene (HCG) and nitrogen-doped graphene (HCNDG) were synthesized with a low-cost and efficient approach. Then, both HCG-based and HCNDG-based electrodes with relatively high specific surface areas were fabricated, and their performance in capacitive deionization were investigated. The results showed that the specific surface area in the crumpled morphology has a key role in determining the electrosorption performance of both of the HCG-based and HCNDG-based electrodes.

### Characterization

**Figure 2:**
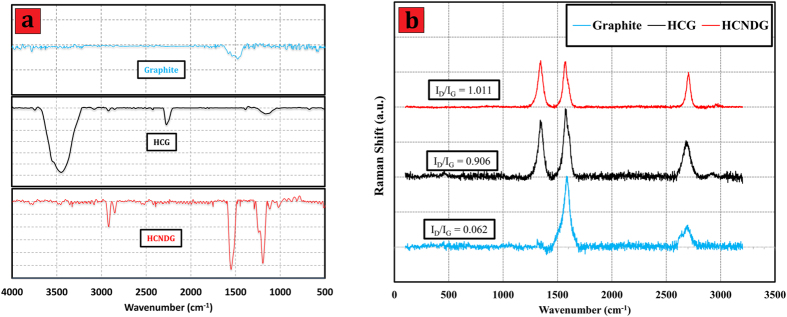
(**a**) FTIR and (**b**) Raman spectra of the pristine graphite, HCG and HCNDG.

Figure 2a presents the Fourier transform infrared spectroscopy (FTIR) spectra of HCG and HCNDG. While there is no cue of functional groups in pristine graphite, it can be seen that both of the HCG-based and HCNDG-based samples illustrate significant peaks in their spectra.

The FT-IR spectrum of HCG shows two new peaks at 1151 and 2268 cm^−1^, which were attributed to the C–N and C≡N stretching vibrations on the HCG generated by the attachment of the -C≡N chain of cyanamide during the diazonium treatment. Also, the peak at 3436 cm^−1^ can be assigned to N-H stretching vibration.

For HCNDG, the peak at 1151 cm^−1^, corresponding to the stretching vibration of C-N, shifted to 1193 cm^−1^. More importantly, the peak at 1550 cm^−1^ was associated with the stretching vibrations of C = N, which provided evidence of the doping of nitrogen atoms into the HCG framework. In addition, two other peaks, one at 2851 cm^−1^ and one at 2921 cm^−1^, are consistent with the sp^3^ C−H and sp^2^ C−H, respectively, which resulted from hydrogenation at the edges and the size reduction. It can be seen that the peaks at 3436 and 2268 cm^−1^, which were attributed to the C≡N and N-H stretching vibrations, respectively, were removed in the FT-IR spectrum of the HCNDG after heating at 900 °C. However, the new peak at 1550 cm^−1^ that was associated with the stretching vibrations of C = N was a suitable substitute for above-mentioned groups.

Raman characterization is a strong measurement for analyzing structure, sp^2^ and sp^3^ hybridized carbon atoms in carbon-based nanomaterials, functionalization, and exfoliation by following alterations in hole and electron doping^30^,^31^,^32^. The Raman spectra of the pristine graphite, HCG, and HCNDG are presented in Fig. 2, panel (b). While the pristine graphite is weak in terms of D intensity, the fairly strong D, G and 2D bands in the HCG and HCNDG samples can be seen at 1351, 1578, and 2699 cm^–1^, respectively. The ratio of the intensities of the D-band to those of the G-band (I_D_/I_G_) was considered to be the amount of disordered carbon (sp^3^-hybridized carbon) relative to graphitic carbon (sp^2^-hybridized carbon). In edge-functionalization and/or nitrogen doping studies of graphene, the higher intensity ratio of I_D_/I_G_ indicates the higher disruption of aromatic π-π electrons, implying partial damage of the graphitic carbon produced by expansion and/or doping^33^. The I_D_/I_G_ ratio of HCG was relatively higher than that of pristine graphite, which confirmed the successful functionalization via a diazonium reaction under microwave irradiation. Also, the Raman results of HCNDG showed a higher I_D_/I_G_ ratio than the pristine graphite as well as HCG, implying that the N-doping procedure was performed. Also, the G and 2D peaks in the spectra of HCG and HCNDG retained their intensities after the diazoniation, which confirmed that the quality of the graphene layers was preserved.

Obviously, Raman spectroscopy can distinguish a single layer from a few layers by focusing on the shape, size, and intensity of the 2D bands^34^,^35^. According to the results of Ferrari *et al.*^34^, as the layer of graphene increases, the 2D band becomes much broader and up-shifted. Accordingly, a noticeable change in sizes, shapes, and intensities of the 2D peaks of HCG and HCNDG are observed clearly as compared with the pristine graphite. It can be seen that the 2D band of the pristine graphite includes a coupled peak, i.e., D1 and D2 peaks, which produced a broad peak^34^,^35^. However, single, sharp 2D peaks were shown in the Raman spectra of HCG and HCNDG. This change in the 2D bands observed in the treated samples potentially verified the presence of single layered- or few layered- graphene and nitrogen-doped graphene.

**Figure 3:**
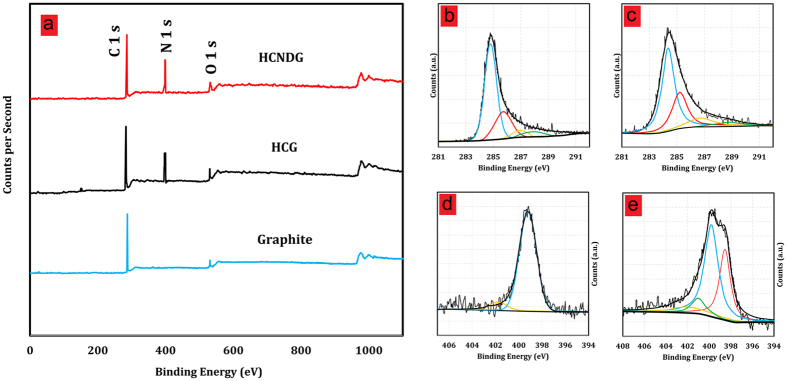
(**a**) XPS survey spectra of pristine graphite, HCG and HCNDG, High-resolution C 1s spectra of (**b**) HCG and (**c**) HGNDG, N1s spectra of (**d**) HCG and (**e**) HGNDG.

The nature and amount of nitrogen-containing groups in pristine graphite, HCG, and HCNDG were studied by X-ray photoelectron spectroscopy (XPS), which are illustrated in Fig. 3a. The survey spectra of HCG and HCNDG have C1s at around 286 eV, N 1s at 399 eV, and O1s at 530 eV. Based on the results, all samples present a very small amount of oxygen, and pristine graphite doesn’t illustrate any nitrogen molecule in its structure. Upon exfoliation and functionalization, the intensity of the N 1s peak increased considerably. The cyanamide functionalities (especially the -C≡N chain) may explain the higher content of nitrogen in the HCG sample. More interestingly, the amount of nitrogen in HCNDG presents a similar result with HCG after thermal treatment in a nitrogen atmosphere. The insignificant decrease in the N component that was obtained after thermal treatment in the nitrogen atmosphere was in agreement with the FTIR, Raman, C 1s, and N 1s results. Figure 3, panels (b) and (c), present the XPS C1s spectra of HCG and HCNDG, respectively.

Both HCG and HCNDG mainly had peaks around 284.6 eV, which corresponded to the C–C/C = C network. Although the intensity of the oxygen element are insignificant, the minor O component in HCG and in HCNDG presents two peaks in the form of the C = O and O–C = O groups at around 286.8 and 288.5 eV, respectively. Also, the C–N peak centered at 285.6 eV was observed in both the HCG and HCNDG spectra.

The N 1s spectrum of HCG (Fig. 3d) composed of two peaks at about 399.2 and 401.8 eV, which would be assigned the bonding configurations of amine functional groups and C≡N or that was detected by FTIR discussed above. However, Fig. 3e (HCNDG spectrum) shows an asymmetric N1s spectrum, which can be decomposed into four peaks, i.e., pyridinic N (~ 398.5 eV), pyrrolic N (~ 399.8 eV), graphitic N (~401 eV), and N-oxides of pyridinic N (~402.1 eV). Interestingly, the functional groups, including the nitrogen groups in HCG, change into more thermally-stable graphitic N after annealing, as illustrated in Fig. 3e. The distributions of the content of N species and their interpretation are presented in Table S1.

**Figure 4:**
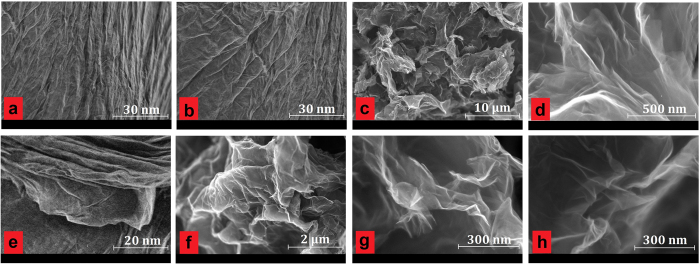
SEM images of (**a–d**) HCG and (**e–h**) HCNDG.

Figure 4a–d show the SEM images of highly-crumpled, few-layered graphene and exfoliated HCG, which were achieved by using a diazonium reaction under microwave irradiation along with physical cracking by a sonicator, demonstrating a noticeable reduction in thickness. The proposed method is able to expand the graphite layers swiftly by initiating a semi-stable diazonium ion and radical reaction with layers. The SEM images of HCG (Fig. 4a–d) showed homogenous, crumpled structures. Such a worm-like surface, with crumpled and curved sheets, was due to strict functionalization and nitrogen impurity. The HCNDG (Fig. 4e–h) also showed the distinct, crumpled structures with 2D geometry, as did HCG. The crumpled structures of HCNDG were attributed to nitrogen doping^8^. More evidence of this is presented by transmission electron microscopy (TEM) and atomic force microscopy (AFM) in the following section.

**Figure 5:**
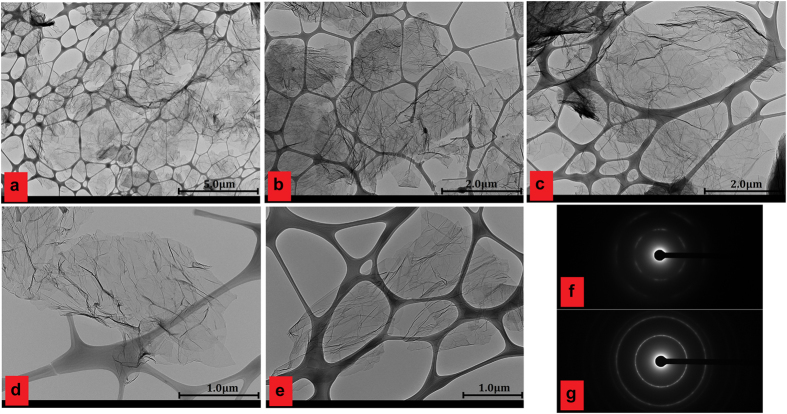
TEM images of (**a** and **b**) HCG, (**c–e**) HCNDG and selected area electron diffraction (SAED) patterns of (**f**) panel a and (**g**) panel c.

Figure 5 shows the TEM images and select area electron diffraction (SAED) patterns of HCG and HCNDG. Figure 5a,b show the TEM images of HCG, in which the crumpled structure can be distinguished. This figure shows a large number of few-layered graphene nanosheets with wrinkled morphology and folded edges. Figure 5c–e show the TEM images of the HCNDG sample, which was comprised of a few-layered graphene with wrinkled morphology and folded edges. A set of highly-crumpled, individual graphene flakes with suitable transparency and without observable graphite crystalline structure in Fig. 5a–e confirmed that these wrinkles resulted from the crumpling of graphene rather than stacking, which is in agreement with the sharp 2D bands in the Raman spectra. As further evidence, the crystalline structures of HCG and HCNDG were verified by selected area electron diffraction (SAED), as shown in Fig. 5f,g, respectively. Selected area electron diffraction (SAED) of both samples illustrated a ring-like diffraction pattern with dispersed bright spots (that were stronger in HCNDG). Such an amorphous structure was attributed partially to the existence of functional groups with abundant defective edges for HCG and nitrogen doping defects for HCNDG, which was in agreement with the high intensity of the D band in the Raman spectra of both samples^36^,^37^. A ring-like diffraction pattern demonstrated the loss of long-range ordering in the sheets^36^.

**Figure 6:**
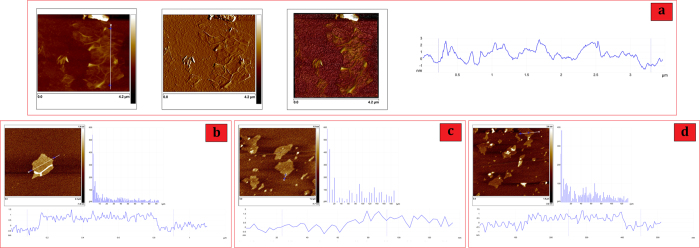
AFM ichnography and cross-section contour of (**a** and **b**) HCNDG and (**c** and **d**) HCG.

AFM was used for the further morphological characterization of the thin-layered HCNDG and for the investigation of the thicknesses of the HCNDG in the final product. AFM samples were prepared by sonicating HCNDG sheets in ethanol for two minutes in a bath sonicator without any additives. Figure 6 shows typical AFM images in which one highly-crumpled sheet was investigated, and, interestingly, the sheets were single-layer sheets, which is in agreement with the sharp 2D bands of the Raman results. As shown in Fig. 6, the HCNDG sheets had thicknesses of about 1 nm, which can be considered to be the thickness of one layer, including the wrinkled morphology.

To investigate the effects of exfoliation and functionalization on the specific surface area of HCG and HCNDG, N_2_ adsorption–desorption isotherms were measured by a surface area analyzer (Quantachrome Autosorb-1 analyzer at 77 K). The N_2_ adsorption–desorption isotherms of the HCG and HCNDG are illustrated in Figure S3 and Table S2. The comparison between the BET results of this study and previous studies^8^,^25^ of graphene materials showed a significant increase in the specific surface area. Also, it can be seen that the N_2_ adsorption amount of the HCNDG was greater than that of HCG. The specific surface area of HCNDG after annealing at the high temperature of 900 °C was 1689 m^2^ g^−1^, but that of HCG was just 1568 m^2^ g^−1^. This lower specific surface area of HCG suggested that the introduction of functional groups between the 2D graphene sheets can fill the porous area. By looking at the dependence of electrosorptive capacity on the specific surface area, the HCG and HCNDG with appropriate surface area can be the suitable candidates for fabricating capacitive deionization electrodes. Note that the highly-crumpled nature of the samples can be an advantageous property for high-capacity ion storage.

### Electrosorption of NaCl

The influences of the characteristics of HCG and HCNDG electrodes with respect to specific surface area and the surface functional groups on electrosorption capacities are investigated in this section. According to previous studies^9^,^11^, the acid treatment phase of carbon nanostructures with increasing specific surface area plays a key role in the preparation of graphene with high electrosorption capacity. Also, when the pristine graphite is exfoliated adequately, it decreases the possibility of severe aggregation, resulting in the high specific surface area. Here, a cost-effective and potentially industrially-scalable method for synthesizing HCG and HCNDG is introduced for preparing samples with significant specific surface area, which plays a key role in the electrosorption capacity of electrodes.

**Figure 7:**
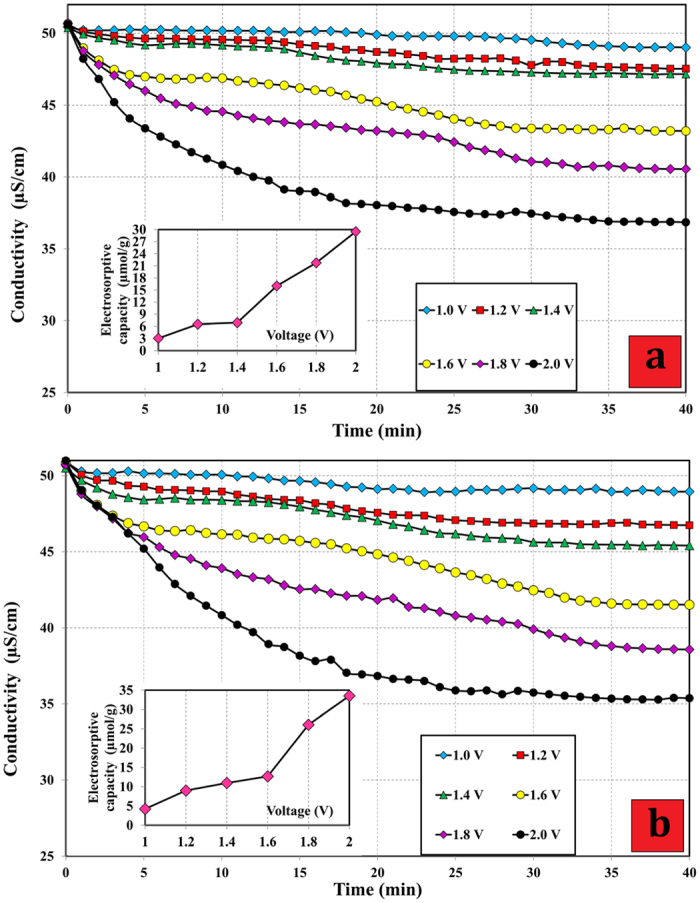
The electrosorption of NaCl onto (**a**) HCG electrode and (**b**) HCNDG electrodes at different applied voltages.

The electrosorption capacity is the amount of ions adsorbed on one gram of the electrode. It commonly is investigated as a function of concentration or conductivity of solutions over the charging procedure. One of the most important parameters that influences the electrosorptive performance of electrodes is the bias potential^11^. As expected, higher voltage means higher electrosorptive capacity. Figure 7a,b illustrate the conductivity transients in solutions and the electrosorptive capacity at various applied voltages for HCG and HCNDG, respectively. Figure 7 illustrates the conductivity transients in solutions during batch-mode experiments at six different applied voltages, i.e., 1.0, 1.2, 1.4, 1.6, 1.8, and 2.0 V. It can be seen that all of the charge processes were performed for 40 min. Obviously, the electrosorptive capacity of both electrodes increased with the directed voltage, and the electrosorptive capacity of HCNDG electrodes was insignificantly larger than that of the HCG electrodes. When the voltage increased from 1.2 to 2.0 V, the electrosorptive capacity of both electrodes increased. It can be seen that at a low voltage of 1.2 V, the decrease of conductivity with time is not obvious. When the applied voltage was greater than 1.2 V, the conductivity obviously decreased with time, indicating that the higher voltage leads to a higher electrosorptive capacity because of the stronger Coulombic interaction^23^. Although the quantity, 2.0 V, is greater than the Nernst potential for the breakdown of water, it should be noted that the intrinsic resistance of the electrodes permits an overvoltage, and no electrolysis took place. In graphene-based electrodes, similar results have been reported by^7^,^9^,^11^,^23^,^24^. As mentioned above, both electrodes show approximately similar trends with excellent capacities in electrosorption of NaCl, although the electrosorptive performance of HGNDG was more than that of the HCG electrodes, which can be attributed to the higher specific surface area. Also, after doping with nitrogen, the transferred electron number increased, implying greater electrical conductivity and the generation an intense electrical double layer for absorbing charged ions from solutions^37^. A special parameter of a relatively high specific surface area can be the most influence factor in enhancing the electrosorption capacity^21^ for graphene-based electrodes. Herein, the HCNDG exhibited higher electrosorption capacity than the HCG-based electrodes in similar experimental conditions. This may be due to the fact that (i) HCNDG itself has a higher specific surface area^11^, (ii) the specific capacitance of electrode material is an important factor for its electrosorption performance^20^, and due to the increased pseudo-capacitive effect of nitrogen doping, HCNDG possesses excellent capacitive performance compared to that of HCG-based materials, and (iii) nitrogen doping can improve the wettability of the interface between the electrolyte and the electrode^27^.

As pioneers in this field with graphene-based electrodes, Xu *et al.*^20^ concluded that the electrochemically-active nitrogen atoms (pyridinic and pyrrolic nitrogen) could simply control local electronic structures, which is beneficial for the improvement of the bonding between the nitrogen atoms and Na^+^ ions in the solution. During this phenomenon, numerous Na^+^ ions are accommodated on the electrode’s surface, including pyridinic and pyrrolic nitrogen, resulting in the possible oxidation/reduction reaction between H_2_O and pyridinic or pyrrolic nitrogen as follows^20^,^27^:


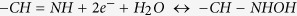


Thus, the wettability of the interface of HCNDG sheets is enhanced, which can be an appropriate parameter in enlarging the available surface area and improving the charge storage of an electrical double layer capacitor^38^,^39^. Therefore, a greater number of Na^+^ ions can be easily accessible to adsorb by HCNDG sheets to form a more effective electrical double layer and accommodated in the pores of the graphene sheets. Moreover, improving the electrical conductivity of the graphene sheets by nitrogen doping may also play a key role in enhancing the electrosorption capacity^26^. Also, as another effective parameter, an increase in the voltage from 1.0 to 2.0 V leads to a sharp increase in the electrosorptive capacity from 3.0547 to 29.4978 μmol/g for HCG and from 4.2124 to 33.5152  μmol/g for HCNDG, which are about 10-fold and 8-fold increases, respectively.

As a result and consistent with previous results^7^,^9^,^11^,^23^,^24^, the applied voltage of 2.0 V did not generate any visible gas bubbles, implying lack of *in situ* water electrolysis. Based on previous results reported by Li *et al.*^11^ accompanied by the above-mentioned reason, the applied voltage of 2.0 V can be the optimum working voltage for HCG-based and HCNDG-based electrodes in terms of electrosorptive capacity and energy consumption. The conductivity at different applied voltages obviously decreases with time. Due to electrolysis, voltages greater than 2.0 V are prohibited in aqueous media.

**Figure 8:**
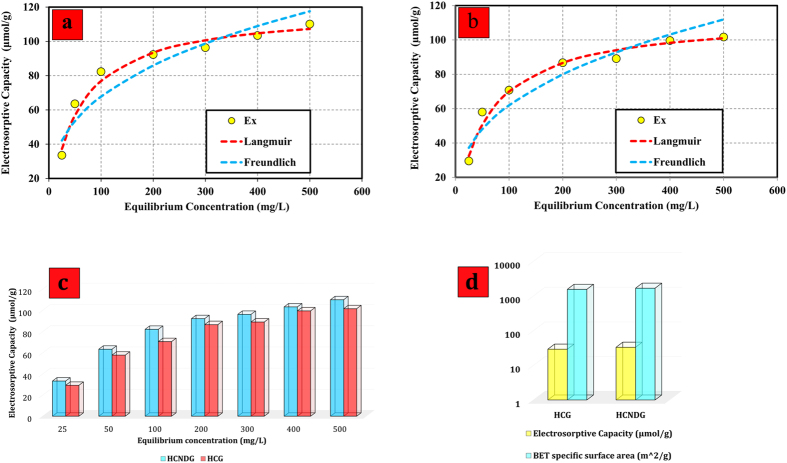
The electrosorption isotherm of NaCl onto (**a**) HCNDG and (**b**) HCG electrodes at 2.0 V, (**c**) a comparison of the electrosorptive capacity of the HCG- and HCNDG-based electrodes at 2.0 V (**d**) a comparison of the surface a and electrosorptive performance of HCG and HCNDG at 2.0 V.

Several electrosorption experiments at 2.0 V and different initial concentrations were conducted to investigate the electrosorptive isotherms of the HCG and HCNDG electrodes. Initial NaCl concentrations of 25, 50, 100, 200, 300, 400, and 500 mg/L were used for both electrodes. Figure 8a,b illustrate the electrosorptive capacities of HCG and HCNDG electrodes, respectively, at 2.0  V. It can be seen that the amount of NaCl adsorbed tends toward zero when the solution tends to be diluted. Figure 8a,b also compare the experimental data with the Langmuir adsorption isotherm model


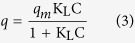


and the Freundlich model


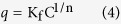


In Eq. (3), C, q, q_m_, and K_L_ are the equilibrium concentration (mg/L), extent of NaCl adsorbed on electrodes (in μg/g of HCG or HCNDG), the maximum amount of adsorption capacity corresponding to the monolayer coverage (μmol/g), and the Langmuir constant (which is associated with the energy of adsorption), respectively. Also, in Eq. (4), K_F_ and 1/n are the Freundlich constant and the tendency of the adsorbate to be adsorbed, respectively.

Table 1 lists the Langmuir and Freundlich parameters and regression coefficients (r^2^). According to the results reported in Fig. 8b and Table 1, the Langmuir isotherm model provided better fits to the experimental data for both the HCG and HCNDG electrodes. It also was observed that the monolayer adsorption was the dominant mechanism in both HCG and HCNDG electrodes during the electrosorption process. As mentioned above, q_m_ is the maximum adsorption based on the Langmuir isotherm model. Thus, for HCG and HCNDG electrodes, the equilibrium electrosorptive capacities are 119.05 and 113.64 (μmol/g), respectively, at the applied voltage of 2.0 V and flow rate of 25  mL/min.

A comparison between the electrosorptive capacity and specific surface area of the HCG-based and HCNDG-based electrodes at different applied voltages and the flow rate of 25 mL/min are depicted in Fig. 8c,d. Both HCNDG and HCG present promising electrosorptive capacities, indicating that the specific surface area plays the most important role in the electrosorptive capacity. Also, consistent with the electrosorption results, the HCNDG provided larger electrosorptive capacity than the HCG. In addition, the gap obtained in the electrosorption results between HCG and HCNDG was attributed to the higher specific surface area and transferred electron number in the presence of N-doping. The transferred electron number means lower charge transfer resistance, implying higher electrosorption capacity^14^. Moreover, previous results^9^ concluded that the conductivity of carbon nanostructures also plays a key role in the electrosorptive process. When a nitrogen atom is doped into graphene, it usually has three common bonding configurations within the carbon lattice, including graphitic, pyridinic, and pyrrolic N. The pyridinic N and pyrrolic N bonds contribute one and two electrons to the π system, respectively, and graphitic N refers to N atoms that are substituted for the C atoms in the hexagonal ring. Clearly, the spin density and charge distribution of carbon atoms can be changed by the adjacent nitrogen dopants^40^, which can induce the “activation site” on the surface of the graphene. Such activated sites are able to participate in the electrosorption. Thus, by looking at the high effective surface area and charge distribution, both HCG and HCNDG have significant potential as candidates in capacitive deionization applications.

Table S3 show a comparison of the electrosorption capacities of various graphene-based electrodes for better understanding. As a comparison with other materials, since active carbon (AC) is the most cost-efficient material as compared with other carbon nanostructures and has a high specific surface area, it is the commonly-used material for the CDI electrodes^24^. Also, AC can be prepared from natural or synthetic sources. Other carbon materials used in CDI research are, for example, ordered mesoporous carbon, carbon aerogels, carbide-derived carbons, carbon nanotubes, and carbon black^41^. Li *et al.*^24^ reported that AC/graphene electrodes have an electrosorption capacity of 0.85 mg/g at the applied voltage of 2.0 V and initial NaCl conductivity of 50 μS/cm. Our results showed the electrosorption capacities of 1.723 and 1.959  mg/g in the presence of HCG- and HCNDG-based electrodes, respectively, at the same experimental conditions.

**Figure 9:**
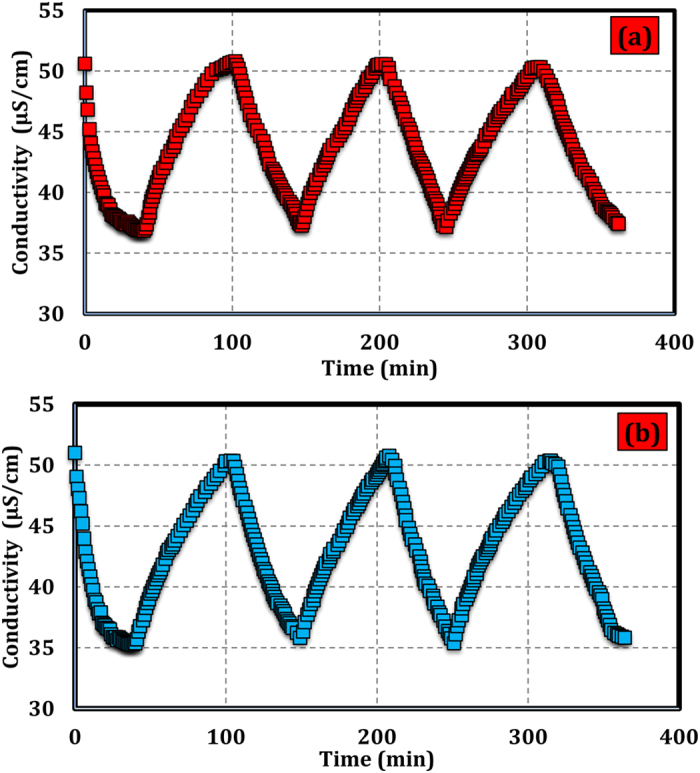
Regeneration of the (**a**) HCG electrode and (**b**) HCNDG electrode in NaCl solution with an initial solution conductivity of 50 μS/cm.

As mentioned above, as the electric field is eliminated, the ions absorbed on the surface of electrodes are effortlessly emancipated. A good regeneration is one of the most important properties for electrode materials. Figure 9 illustrates the electrosorption–desorption cycles of the HCG and HCNDG electrodes, which are conducted by repeating several charging (2.0 V) and discharging (0  V) processes in NaCl solution with an initial conductivity of around 51  μS/cm. Note that regeneration of HCG and HCNDG electrodes and repeatability of the electrosorption process were realized in the unit cell. The electrosorption capacity decline of electrodes have not been observed in the unit cell after over 3 charge–discharge cycles, which is in agreement with recent studies^9^,^11^,^42^.

## Conclusions

An industrially-scalable, cost-effective, and simple approach was used to synthesize HCG and HCNDG with large specific surface area. Highly-crumpled graphene and highly-crumpled nitrogen-doped graphene with appropriate specific surface area were used as the electrodes for capacitive deionization that showed considerable potential. The results suggested that the electrosorption performances of both electrodes were very high, and they are well-suited for capacitive deionization applications. The study also suggested that the Langmuir isotherm model would provide better fits of the experimental data for both electrodes, implying that the monolayer adsorption on the electrodes was the key mechanism. Also, in the presence of HCNDG and HCG, respectively, high electrosorption capacities of 29.4978 and 33.5152 µmol/g and high specific surface area of 1689 and 1568 m^2^/g illustrated that highly-crumpled materials can be the best options for a new generation of capacitive deionization devices.

## Additional Information

**How to cite this article**: Amiri, A. *et al.* Microwave-Assisted Synthesis of Highly-Crumpled, Few-Layered Graphene and Nitrogen-Doped Graphene for Use as High-Performance Electrodes in Capacitive Deionization. *Sci. Rep.* **5**, 17503; doi: 10.1038/srep17503 (2015).

## Supplementary Material

Supplementary Information

## Figures and Tables

**Table 1:** The isotherms parameters of NaCl adsorption/electrosorption on HCG and HCNDG.

Electrode	Isotherm	Model Equation	Parameter	value
**HCG**	Langmuir	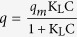	q_m_	113.64
			K_L_	0.016
			R^2^	0.996
**HGC**	Freundlich	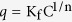	K_f_	11.391
			n	2.7196
			R^2^	0.8902
**HCNDG**	Langmuir	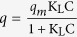	q_m_	119.05
			K_L_	0.0182
			R^2^	0.995
**HCNDG**	Freundlich	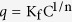	K_f_	13.987
			n	2.9019
			R^2^	0.8712
