# Supplementary material for: Microwave-Assisted Synthesis of Highly-Crumpled, Few-Layered Graphene and Nitrogen-Doped Graphene for Use as High-Performance Electrodes in Capacitive Deionization
Source: Sci Rep. 2015 Dec 8;5:17503. doi: 10.1038/srep17503 (PMC4672334; doi:10.1038/srep17503)
Supplement: Supplementary Information [file srep17503-s1.pdf]

Supplementary Information

Microwave-Assisted Synthesis of Highly-Crumpled, Few-Layered Graphene and Nitrogen-Doped Graphene for Use as High-Performance Electrodes in Capacitive Deionization

Ahmad Amiri [1, †], Goodarz Ahmadi [2,*], Mehdi Shanbedi [3], Maryam Savari [4], S.N. Kazi [5, ‡], B.T. Chew [6]

[1, †] Department of Mechanical Engineering, University of Malaya, Kuala Lumpur, Malaysia, Corresponding author and E-mail addresses: ahm.amiri@gmail.com and ahm.amiri@siswa.um.edu.my

[2,*] Professor, Department of Mechanical and Aeronautical Engineering, Clarkson University, Potsdam, NY 13699, USA. Corresponding author and E-mail addresses: gahmadi@clarkson.edu

[3] Department of Chemical Engineering, Faculty of Engineering, Ferdowsi University of Mashhad, Mashhad, Iran, Email: mehdi.shanbedi@stu-mail.um.ac.ir

[4] Faculty of Computer Science and Information Technology, University of Malaya, Kuala Lumpur, Malaysia. E-mail addresses: maryamsavari@siswa.um.edu.my

[5, ‡] Senior Lecturer, Department of Mechanical Engineering, University of Malaya, Kuala Lumpur, Malaysia. Corresponding author and E-mail addresses: salimnewaz@um.edu.my

[6] Senior Lecturer, Department of Mechanical Engineering, University of Malaya, Kuala Lumpur, Malaysia. E-mail addresses: chewbeeteng@um.edu.my

Chemicals.

Chemical materials and solvents were obtained from Sigma-Aldrich in analytical grade without future purification. Graphite (purity of 99.9%) was received from Alfa Aesar and applied without extra purification. Also, a Millipore PTFE membrane (pore size of 0.45 μm) was purchased from Whatman Co.

Schematic procedure of exfoliation of graphite flakes to HCG and HCNDG with cyanamide is depicted in Figure S_1. Mechanism for the graphite exfoliation and/or HCG/HCNDG preparation includes generation of semi-stable diazonium ion, which then initiates a radical reaction with flakes. Then treated graphite with higher dispersibility in DMF open a new gateway for expanding graphite to graphene flakes by placing between layers. In a typical experiment, pristine graphite (10 mg) and deionized water (30 mL) were poured into a 100 mL vessel. The reaction vessel was sonicated for 5 minutes and the cyanamide (4 equiv per graphite) and isoamyl nitrite (2 equiv per graphite) were added during sonication time, then poured into a Teflon vessel and placed in a microwave chamber for 15 min at 700W. The mixture was then mixed vigorously on a stirrer at 80 ºC overnight. The obtained product was followed by cooling to the room temperature, filtered on a PTFE filter and washed with deionized water, methanol and acetone. Thus, a semi-stable diazonium ion produced and then resulted in a radical reaction with graphite flakes. The product was removed from the filter and sonicated for 1 hr in DMF. The resulting black ink-like dispersion was left to sit 24 hrs to separate large unstable graphite aggregates. The dispersed cyanamide-treated graphene was collected by a low speed centrifugation of 1000 rpm for 30 min. The filtrated supernatant dried and denoted as HCG, comprising graphene flakes was reserved for further treatment. To prepare HCNDG, the resulting HCG materials was then placed to a furnace under nitrogen atmosphere and heated at 900 ºC to decompose the functionality including amine group into the nitrogen-containing species such as $C_2N_2^+$, $C_3N_2^+$, $C_3N_3^+$ [1, 2, 3], that prepare nitrogen sources for changing HCG into HCNDG.

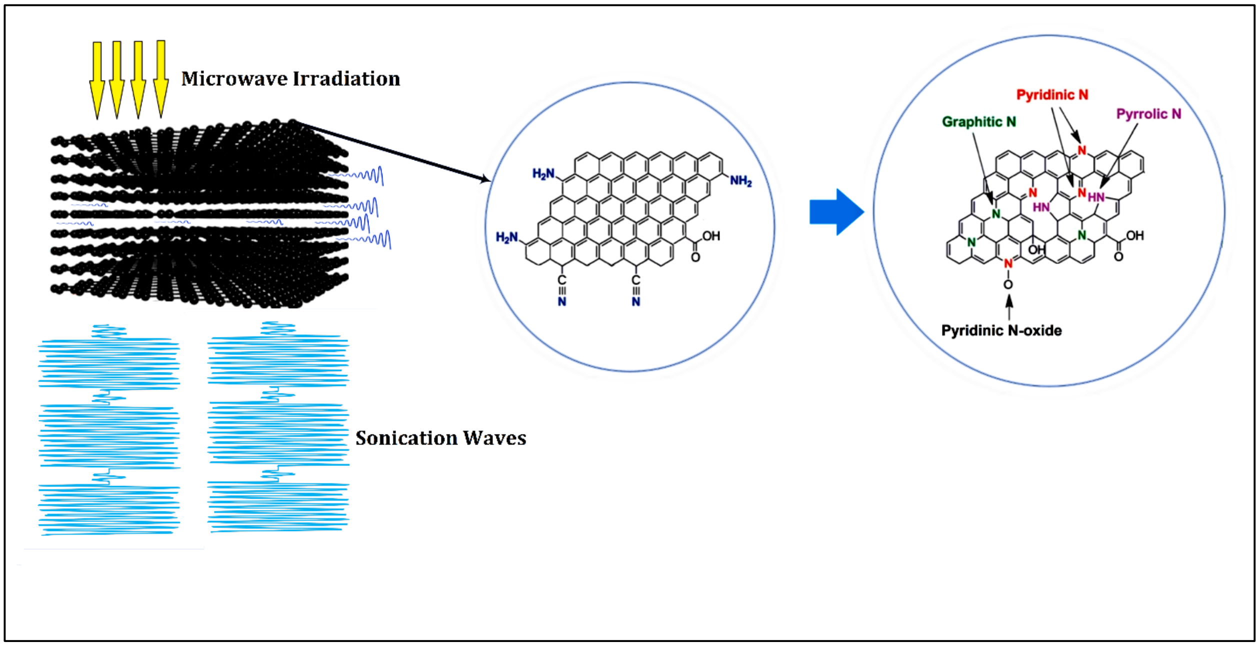

Figure S1. The experimental procedure for exfoliation of graphite and synthesizing HCG and HCNDG.

Characterizations.

The infrared spectra of samples were studied by Fourier transform infrared spectroscopy (Bruker IFS 66/S) in the region of 400–4000 cm^{-1} at room temperature (around 25 ˚C), and all samples were placed on KBr pellets. To investigate functionalization and explore alterations in the layers, Raman spectra were obtained via a Renishaw confocal spectrometer at 514 nm at room temperature (around 25 ˚C).The thermogravimetric analyses (TGA-50 Shimadzu) is performed at the nitrogen atmosphere. The weight losses of HCG and HCNDG samples in the TGA analyzer was recorded at heating rate of 10 ˚ C /min.

In addition, transmission electron microscopy (HT7700, High-Contrast/High-Resolution Digital TEM), and scanning electron microscopy (Cold-Emission FE-SEM SU8000, Series UHR) were employed to study surface morphology of HCG and HCNDG samples. As a TEM sample, each treated HCG and HCNDG powder was first dispersed in ethanol, dropped on a lacey carbon grid and subsequently dried under vacuum. An ultrasonic processor (Misonix Inc., Farmingdale, New York, NY, USA) with output of 600 W was used. All samples sonicated with 85% of total power of ultrasonic processor. Also, a Milestone MicroSYNTH programmable

microwave system equipped with temperature controller was employed to perform the process of functionalization.

To investigate more in morphology of HCG and HCNDG samples, atomic force microscopy, AFM, (Bruker) were utilized. AFM observation was conducted for the slightly sonicated samples on the freshly cleaved mica surfaces (ScanAsyst mode, frequency 1 Hz, Bruker).

Experiment Validation.

Figure S2 illustrates a correlation which results from a relationship of solution conductivity (µs/cm) and concentration (mg/L).

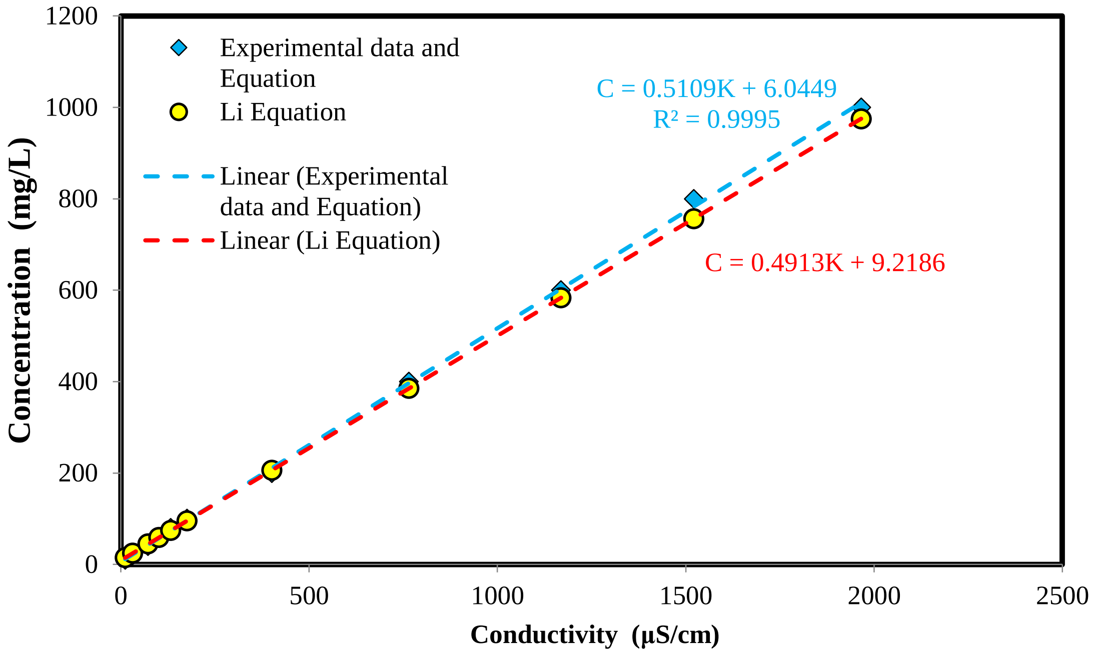

Figure S2. The relationship between conductivity and solution concentration as compared with Li et al. [4].

The characterization of HGNDG and HCG samples including the content distributions of N species are presented in Table S1. The elemental analysis showed that the nitrogen weight content in the resulting HCG and after annealing (HCNDG) at 900 ºC were 17.57% N and 10.32 % N, respectively.

Table S1. The content distributions of N species in HCG and HCNDG.

Materials	C%	N%	N/C	Pyridinic N	Pyrrolic N	Graphitic N	N-oxides of pyridinic N
**HCG**	69.13	17.56	0.254	---	---	---	---
**HCNDG**	79.75	10.32	0.129	4.12	4.72	0.81	0.67

N_2 adsorption–desorption

The N_2 adsorption–desorption isotherms of the pristine graphite, HCG and HCNDG are shown in Figure S3 and Table S2. It can be seen that the N_2 adsorption amount of the HCNDG was higher than that of HCG.

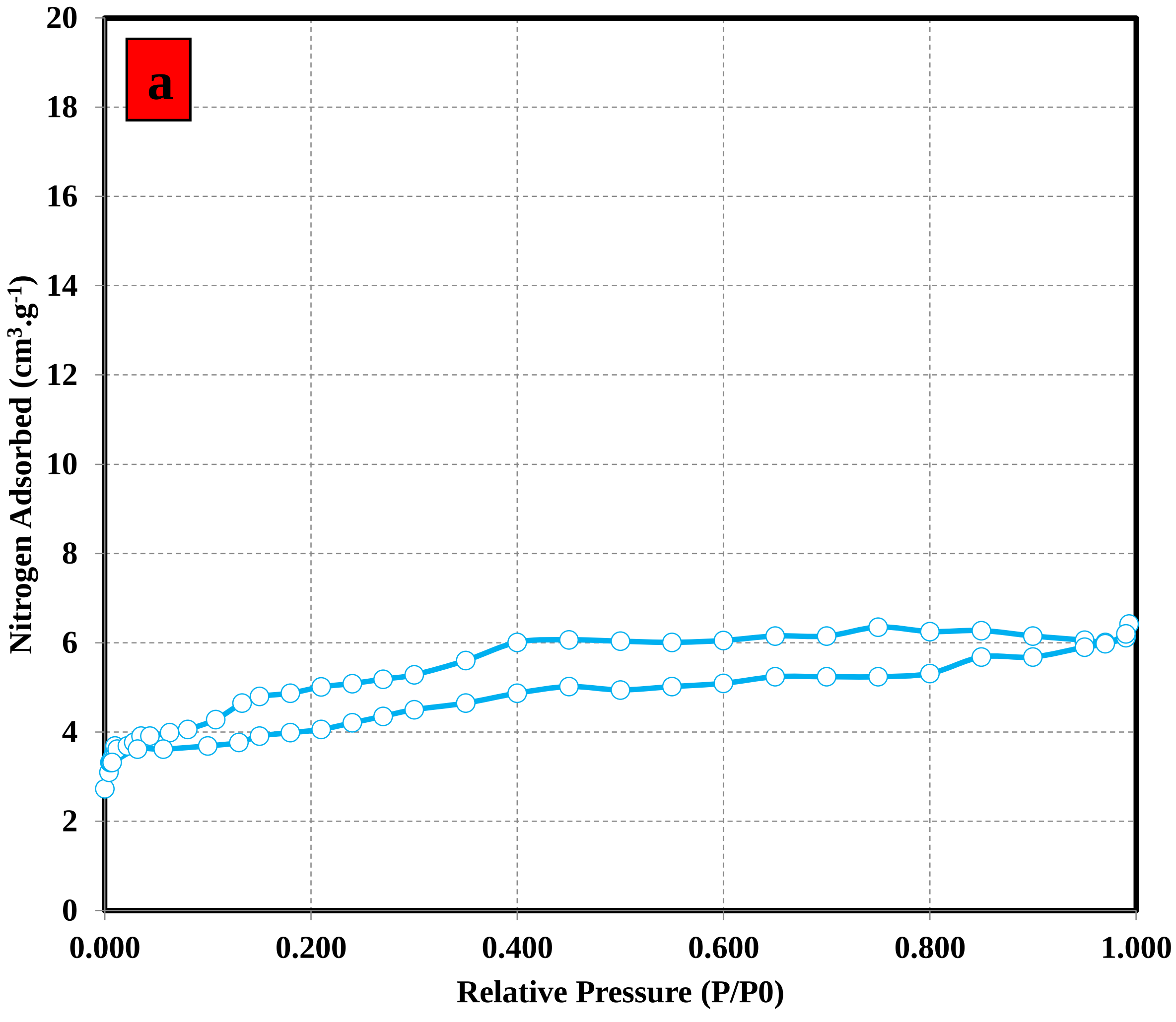

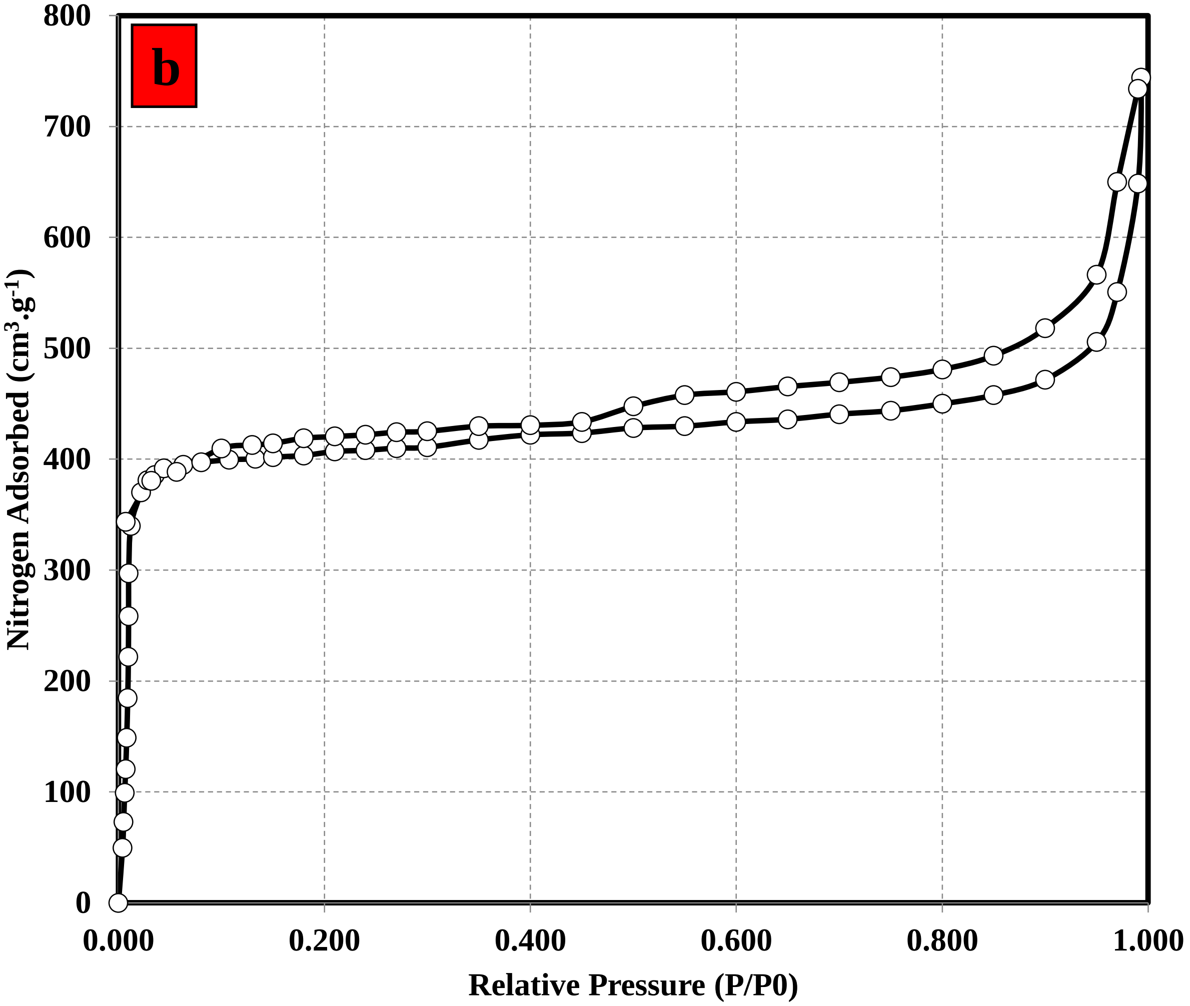
b
Nitrogen Adsorbed (cm3.g-1)
800
700
600
500
400
300
200
100
0
0.000
0.200
0.400
0.600
0.800
1.000
Relative Pressure (P/P0)

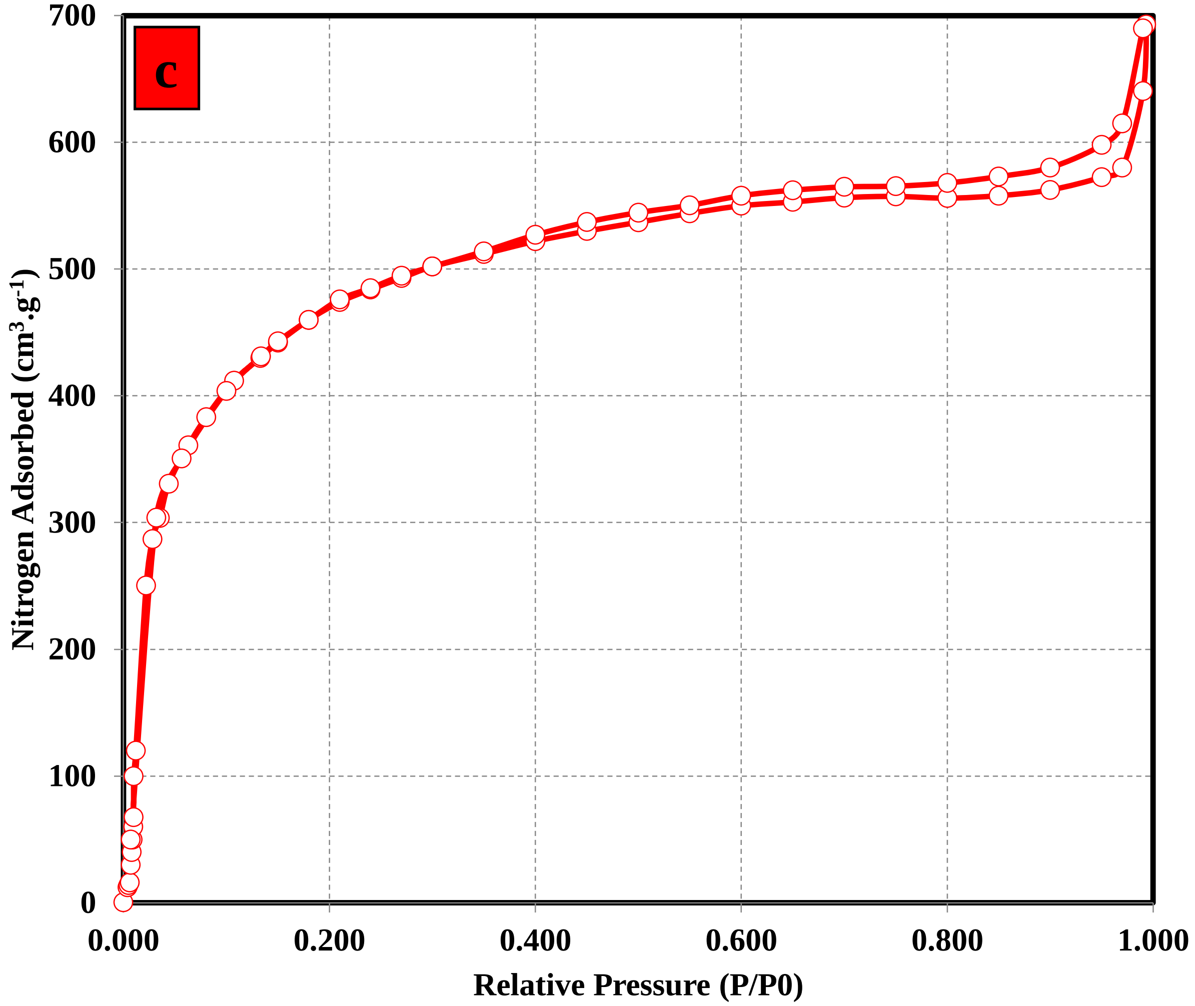

Figure S3. N_2-adsorption/desorption of (a) the pristine graphite, (b) HCG and (c) HCNDG.

Table S2. Pore structure of the HCG and HCNDG.

Sample	S_{BET} (m^2 g^{-1})	Total pore volume (cm^3 g^{-1})
**HCG**	1568	1.33
**HCNDG**	1689	1.39

Comparison

Table S3 show a comparison of the electrosorption capacities of various graphene-based electrodes. The electrosorption capacities of HCG and HCNDG were compared with those of other graphene-based electrode material reported in the literature. Obviously, both samples had a relatively high electrosorption capacity among these graphene-based electrodes in similar experimental conditions, in particular, HCNDG showed the highest amount of electrosorption capacity.

Table S3. The electrosorption capacities of various graphene-based electrodes.

Sample	Applied Voltage (V)	Initial NaCl Conductivity (μS cm^{-1})	Electrosorption Capacity (mg g^{-1})
Graphene [5]	2.0	50	1.85
Pyridine–thermal prepared graphene [6]	2.0	87	0.88
Graphene-like nanoflakes [4]	2.0	57	1.36
Graphene/CNTs [7]	1.6	100	0.88
Graphene/MC [8]	2.0	89.5	0.73
Graphene/AC [9]	2.0	50	0.85
HCG (this work)	2.0	50	1.723
HCNDG (this work)	2.0	50	1.959

References

1. Fischer A, Müller JO, Antonietti M, Thomas A. Synthesis of ternary metal nitride nanoparticles using mesoporous carbon nitride as reactive template. *ACS nano* **2**, 2489-2496 (2008).

2. Wen Z, *et al.* Crumpled Nitrogen-Doped Graphene Nanosheets with Ultrahigh Pore Volume for High-Performance Supercapacitor. *Advanced Materials* **24**, 5610-5616 (2012).

3. Fischer A, Antonietti M, Thomas A. Growth confined by the nitrogen source: synthesis of pure metal nitride nanoparticles in mesoporous graphitic carbon nitride. *Advanced Materials* **19**, 264-267 (2007).

4. Li H, Zou L, Pan L, Sun Z. Novel graphene-like electrodes for capacitive deionization. *Environmental science & technology* **44**, 8692-8697 (2010).

5. Li H, Lu T, Pan L, Zhang Y, Sun Z. Electrosorption behavior of graphene in NaCl solutions. *Journal of Materials Chemistry* **19**, 6773-6779 (2009).

6. Wang H, Zhang D, Yan T, Wen X, Shi L, Zhang J. Graphene prepared via a novel pyridine–thermal strategy for capacitive deionization. *Journal of Materials Chemistry* **22**, 23745-23748 (2012).

7. Li H, Liang S, Li J, He L. The capacitive deionization behaviour of a carbon nanotube and reduced graphene oxide composite. *Journal of Materials Chemistry A* **1**, 6335-6341 (2013).

8. Zhang D, Wen X, Shi L, Yan T, Zhang J. Enhanced capacitive deionization of graphene/mesoporous carbon composites. *Nanoscale* **4**, 5440-5446 (2012).

9. Li H, Pan L, Nie C, Liu Y, Sun Z. Reduced graphene oxide and activated carbon composites for capacitive deionization. *Journal of Materials Chemistry* **22**, 15556-15561 (2012).
